# Molecular Mechanisms of Short-Term Plasticity: Role of Synapsin Phosphorylation in Augmentation and Potentiation of Spontaneous Glutamate Release

**DOI:** 10.3389/fnsyn.2018.00033

**Published:** 2018-10-30

**Authors:** Qing Cheng, Sang-Ho Song, George J. Augustine

**Affiliations:** ^1^Laboratory of Neurobiology, National Institute of Environmental Health Sciences, National Institutes of Health, Durham, NC, United States; ^2^Lee Kong Chian School of Medicine, Nanyang Technological University, Singapore, Singapore; ^3^Institute of Molecular and Cell Biology, Singapore, Singapore

**Keywords:** post-tetanic potentiation, synapsins, neurotransmitter release, PKA, synaptic plasticity

## Abstract

We used genetic and pharmacological approaches to identify the signaling pathways involved in augmentation and potentiation, two forms of activity dependent, short-term synaptic plasticity that enhance neurotransmitter release. Trains of presynaptic action potentials produced a robust increase in the frequency of miniature excitatory postsynaptic currents (mEPSCs). Following the end of the stimulus, mEPSC frequency followed a bi-exponential decay back to basal levels. The time constants of decay identified these two exponential components as the decay of augmentation and potentiation, respectively. Augmentation increased mEPSC frequency by 9.3-fold, while potentiation increased mEPSC frequency by 2.4-fold. In synapsin triple-knockout (TKO) neurons, augmentation was reduced by 83% and potentiation was reduced by 74%, suggesting that synapsins are key signaling elements in both forms of plasticity. To examine the synapsin isoforms involved, we expressed individual synapsin isoforms in TKO neurons. While synapsin IIIa rescued both augmentation and potentiation, none of the other synapsin isoforms produced statistically significant amounts of rescue. To determine the involvement of protein kinases in these two forms of short-term plasticity, we examined the effects of inhibitors of protein kinases A (PKA) and C (PKC). While inhibition of PKC had little effect, PKA inhibition reduced augmentation by 76% and potentiation by 60%. Further, elevation of intracellular cAMP concentration, by either forskolin or IBMX, greatly increased mEPSC frequency and occluded the amount of augmentation and potentiation evoked by electrical stimulation. Finally, mutating a PKA phosphorylation site to non-phosphorylatable alanine largely abolished the ability of synapsin IIIa to rescue both augmentation and potentiation. Together, these results indicate that PKA activation is required for both augmentation and potentiation of spontaneous neurotransmitter release and that PKA-mediated phosphorylation of synapsin IIIa underlies both forms of presynaptic short-term plasticity.

## Introduction

Numerous forms of activity-dependent synaptic plasticity enable dynamic changes in the properties of neural circuits (Zucker and Regehr, 2002; Abbott and Regehr, 2004; Jackman and Regehr, 2017; Nicoll, 2017). Bouts of high-frequency synaptic activity generate augmentation and potentiation (often called post-tetanic potentiation, or PTP), two forms of short-term plasticity that enhance neurotransmitter release for tens of seconds to minutes (Magleby and Zengel, 1976a,b; Fioravante and Regehr, 2011; Regehr, 2012). Augmentation and potentiation apparently enhance neurotransmitter release via a variety of presynaptic mechanisms, including increasing quantal release probability (Kalkstein and Magleby, 2004; Zhao and Klein, 2004; Korogod et al., 2007; Lee et al., 2008; Valente et al., 2012), enhancing the readily releasable pool of synaptic vesicles (Zhao and Klein, 2004; Habets and Borst, 2007; Lee et al., 2008; Valente et al., 2012) and/or other mechanisms (Habets and Borst, 2006; Humeau et al., 2007; Korogod et al., 2007; Neher and Sakaba, 2008; He et al., 2009).

It is well established that both augmentation and potentiation are triggered by a transient rise in calcium concentration within the presynaptic terminal (Erulkar and Rahamimoff, 1978; Kretz et al., 1982; Swandulla et al., 1991; Delaney and Tank, 1994; Kamiya and Zucker, 1994; Regehr et al., 1994; Brager et al., 2003; Kalkstein and Magleby, 2004; Habets and Borst, 2005; Korogod et al., 2005). However, the downstream effectors within these activity-dependent calcium signaling pathways remain unclear. For the case of augmentation, studies have indicated an important role for munc13, a calcium-sensitive regulator of the SNARE proteins that mediate neurotransmitter release (Rosenmund et al., 2002; Gioia et al., 2016). In contrast, a variety of calcium-regulated protein kinase pathways have been implicated in potentiation (Alle et al., 2001; Brager et al., 2002; Sweatt, 2004; Korogod et al., 2007; Fioravante et al., 2011; Lee et al., 2010). Among these, protein kinases C (PKC) and A (PKA) have received the most attention. Substantial evidence indicates that PKC activity is required for potentiation at the calyx of Held synapse (Korogod et al., 2007; Fioravante et al., 2011). However, potentiation at other synapses is independent of PKC (Wang et al., 2016). At still other synapses, inhibiting PKA activity prevents potentiation, also suggesting a role for this protein kinase in potentiation (Alle et al., 2001; Valente et al., 2012).

The downstream targets of these protein kinases are also unclear. Members of the synapsin gene family are leading candidates. Synapsins are a family of vesicle-associated proteins, encoded by three genes, that regulate synaptic vesicle dynamics and neurotransmitter release (Greengard et al., 1993; Rosahl et al., 1995; Cesca et al., 2010; Song and Augustine, 2015). Synapsins are substrates of several protein kinases, including PKA, calcium-calmodulin regulated protein kinases and the mitogen-activated protein kinase (Jovanovic et al., 1996; Jovanovic et al., 2001; Chi et al., 2003; Kohansal-Nodehi et al., 2016). Therefore, synapsins could serve as downstream effectors to regulate neurotransmitter release during potentiation. Indeed, knock-out of the synapsin I gene partially reduces potentiation in cultured hippocampal neurons (Valente et al., 2012), while deletion of both synapsin I and II genes reduces potentiation in the hippocampus (Rosahl et al., 1995) and injection of anti-synapsin antibodies reduces potentiation at *Aplysia* synapses (Humeau et al., 2001). These studies suggest that synapsins and their phosphorylation play an important role in potentiation.

Here we have done experiments in cultured hippocampal neurons to clarify the roles of protein kinases and synapsins in synaptic augmentation and potentiation. Pharmacological experiments indicate that PKA is important for both augmentation and potentiation of spontaneous glutamate release at excitatory synapses. Synapsins also are important because augmentation and potentiation are greatly reduced by knock-out of all three synapsin genes. Further, synapsins apparently are the main substrates of PKA because mutation of a PKA phosphorylation site in synapsin IIIa largely abolished the ability of this isoform to rescue augmentation and potentiation in synapsin knock-out neurons. Our results lead to a new model for the signaling pathways involved in these two forms of short-term plasticity.

## Materials and Methods

### Hippocampal Neuronal Cultures

Homozygous synapsin triple-knockout (TKO) mice and matching triple wild-type (TWT) mice were produced as described previously (Gitler et al., 2004a,b). The procedures used to maintain and use these mice were approved by our institutional Animal Care and Use Committees. Newborn pups (postnatal day 0–1) were used to prepare dissociated hippocampal neurons. Microisland cultures were prepared from these neurons as described in Bekkers and Stevens (1991), with the addition of glia feeder cells to promote neuronal survival. Neurons were allowed to mature for 10–14 days before being used for electrophysiological recordings.

### Electrophysiological Data Acquisition and Analysis

To record spontaneous miniature excitatory postsynaptic currents (mEPSCs), whole-cell patch-clamp recordings were made from single neurons on microislands (Gitler et al., 2004a). Patch pipettes (4–6 MOhm) were filled with intracellular solution containing (in mM): 50 K-glutamate, 71 K-gluconate (Fluka, Buchs, Switzerland), 15 NaCl, 6 MgCl_2_, 0.5 EGTA, 5 Na_2_ATP, 0.3 Na_2_GTP, and 20 HEPES-KOH, pH 7.3 (285 mOsm). The extracellular solution contained (in mM): 150 NaCl, 3 KCl, 2 CaCl_2_, 2 MgCl_2_, 20 glucose, and 10 HEPES-NaOH, pH 7.3 (310 mOsm). All materials were from Sigma, unless specified otherwise. An EPC-9D amplifier (HEKA, Lambrecht/Pfalz, Germany) was used to voltage clamp neurons at a holding potential of −70 mV. Under these conditions, spontaneous EPCSs are solely due to mEPSCs that were blocked by the AMPA receptor antagonist, CNQX (20 μM). Spontaneous synaptic events were first detected automatically, with an amplitude threshold of 8 pA, using the MiniAnalysis program (Synaptosoft, Decatur, GA, United States), and then subsequently manually screened to remove any residual artifacts. mEPSC frequency was measured within 5 s bins.

**FIGURE 1:**
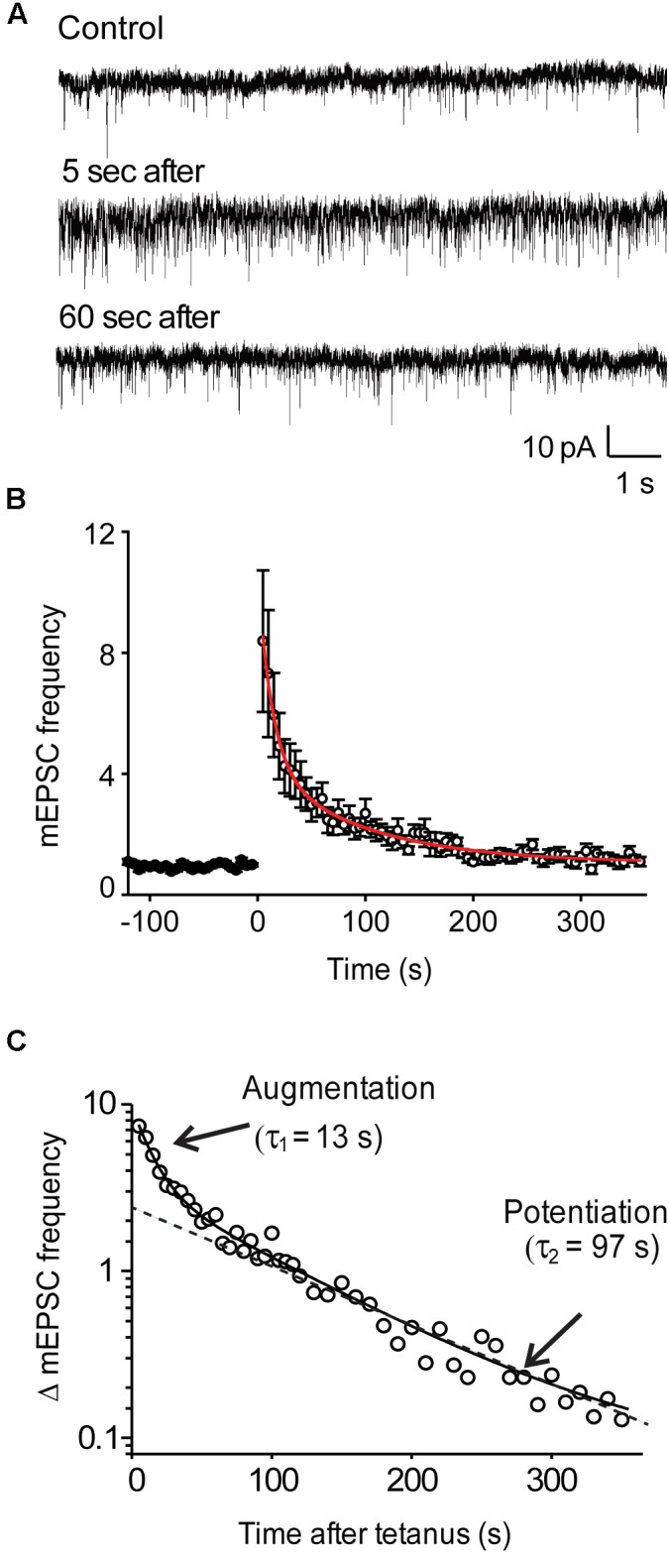
Activity-dependent enhancement of miniature EPSC frequency in wild-type hippocampal neurons. **(A)** Representative recordings of mEPSCs before, as well as 5 and 60 s after the end of a stimulus train (50 Hz, 2 s). **(B)** Time course of mEPSC frequency changes produced by stimulation (at time = 0). Points indicate mean values of mEPSC frequency (*n* = 9), normalized to baseline values measured prior to the stimulus train, and error bars indicate ± SEM. Red curve indicates fit of bi-exponential decay function. **(C)** Semi-logarithmic plot of the post-tetanic changes in mEPSC frequency shown in panel **(B)**. Solid curve indicates bi-exponential decay function, while dashed line indicates time course of slower potentiation component.

“Presynaptic” action potentials were evoked by using the recording pipette to depolarize the neuron to +40 mV for 0.5 ms. To measure the amplitudes of augmentation and potentiation evoked by a train of such stimuli (50 Hz, 2 s), we first normalized the response by dividing mEPSC frequency at each time point following the stimulus train by the basal frequency of mEPSCs prior to the stimulus (as in Figure 1B). We then fitted the normalized mEPSC frequency for each timepoint, *t*, with a 2-exponential decay function:

$$f(t)=A_{1}e^{\frac{-t}{{\text{τ}}_{1}}}+A_{2}e^{\frac{-t}{{\text{τ}}_{2}}}+f_{0}$$

where A_1_ represents the amplitude of augmentation and A_2_ represents the amplitude of potentiation, τ_1_ is the time constant of augmentation and τ_2_ is the time constant of potentiation, and f_0_ is the mean baseline frequency of mEPSCs. Using this equation, the two components were mathematically separated and the contributions of each component were independently defined. Thus, the A_1_ and A_2_ amplitude values reflect the increases in mEPSC frequency independently contributed by each process.

Photoactivation of opto-Gs (Airan et al., 2009) was done with blue light (470 ± 20 nm) from a mercury lamp, with light flash duration controlled by an electronic shutter (Uniblitz).

Differences between experimental parameters measured in two groups were tested for statistical significance using the Student’s *t*-test. For comparisons across more than two experimental groups, we first performed a normality test (Kolmogorov–Smirnov test) to determine whether the data were normally distributed. All datasets were found to be normally distributed, permitting the use of parametric statistical tests. Specifically, data were analyzed by using a one-way ANOVA to determine whether there were any significant differences between groups, followed by the *post hoc* Holm–Bonferroni method to control for the familywise error rate associated with multiple comparisons. Throughout the “Results” section, the outcome of these statistical analyses are reported as both t and *p*-values.

### Viral Expression of Synapsin Isoforms

EGFP-tagged synapsin Ia, Ib, IIa, IIb, and IIIa were subcloned into a pFUGW shuttle vector, where the inserted synapsin genes were driven by the human polyubiquitin-C promoter. Site-directed mutagenesis was done using Quikchange kit (Stratagene). Lentivirus was then prepared as described in Lois et al. (2002). Opto-β2-AR plasmid (Airan et al., 2009) was a generous gift from Dr. K Deisseroth. Neurons were infected after 3–4 days in culture, with a 1:3 multiplicity of infection, and studied 7–10 days post-infection. Song and Augustine (2016) have reported that cultured TKO neurons virally infected with various synapsin constructs express exogenous synapsin isoforms 1.3 to 2.5-fold greater than the expression of endogenous synapsins in TWT neurons, indicating mild overexpression in virally infected TKO neurons. Electrical recordings were made only from neurons that were infected, based on visible expression of GFP-tagged synapsins. Thus, 100% of the “presynaptic” cells that we stimulated were transfected.

## Results

Our experiments measured augmentation and potentiation of spontaneous transmitter release at excitatory synapses of microisland-cultured hippocampal neurons. The advantage of measuring synaptic plasticity via spontaneous release, rather than by measuring release evoked by presynaptic action potentials, is that this approach circumvents several confounds – such as activity-dependent changes in quantal size (He et al., 2009; Fioravante et al., 2011), presynaptic action potential waveform (Habets and Borst, 2005), or presynaptic calcium currents (Habets and Borst, 2006) – that make it difficult to interpret measurements of action-potential evoked synaptic responses. Previous work has established that the kinetics of synaptic augmentation and potentiation of spontaneous transmitter release are very similar to the kinetics of action-potential evoked transmitter release (Erulkar and Rahamimoff, 1978; Zengel and Magleby, 1981; Eliot et al., 1994).

### Augmentation and Potentiation of Spontaneous Transmitter Release

When recording synaptic responses, the rapid kinetics of the autaptic excitatory postsynaptic currents (EPSCs) evoked by brief depolarizations could be used to identify glutamatergic synapses (Gitler et al., 2004a). To measure the rate of spontaneous glutamate release at these excitatory synapses, we monitored the frequency of mEPSCs before and after trains of depolarizing stimuli (50 Hz, 2 s). Application of such tetanic stimuli produced a robust increase in the frequency of mEPSCs in wild-type neurons (Figure 1A). Following the end of the stimulus, mEPSC frequency declined back to baseline levels over a few hundred seconds (Figure 1B). This post-tetanic decay of mEPSC frequency could be fit with the sum of two exponential functions (red line in Figure 1B), one with a time constant of 13.2 ± 1.7 s (*n* = 9) and a second with a slower time constant of 97.1 ± 15.6 s (*n* = 9). These time constants identify these two components as the decay of augmentation and potentiation (Zengel and Magleby, 1982; Regehr, 2012). The bi-exponential decay of mEPSC frequency after a tetanus was readily visualized when plotted on semi-logarithmic coordinates, making both the decay of augmentation and potentiation appear linear (Figure 1C). The augmentation component increased mEPSC frequency by 931% ± 221% (*n* = 7), while the potentiation component increased mEPSC frequency by 245% ± 140% (*n* = 7).

### Synapsins Are Required for Augmentation and Potentiation

Synapsins are the most abundant phosphoprotein in the brain (Hilfiker et al., 1999, 2005) and are known to be phosphorylated after tetanic stimuli and other forms of prolonged depolarization (Greengard et al., 1993; Kohansal-Nodehi et al., 2016). Given previous evidence indicating a role for both synapsins and protein phosphorylation in potentiation (see references in “Introduction” section), we hypothesized that synapsins could play a role in short-term plasticity by serving as protein kinase substrates.

**FIGURE 2:**
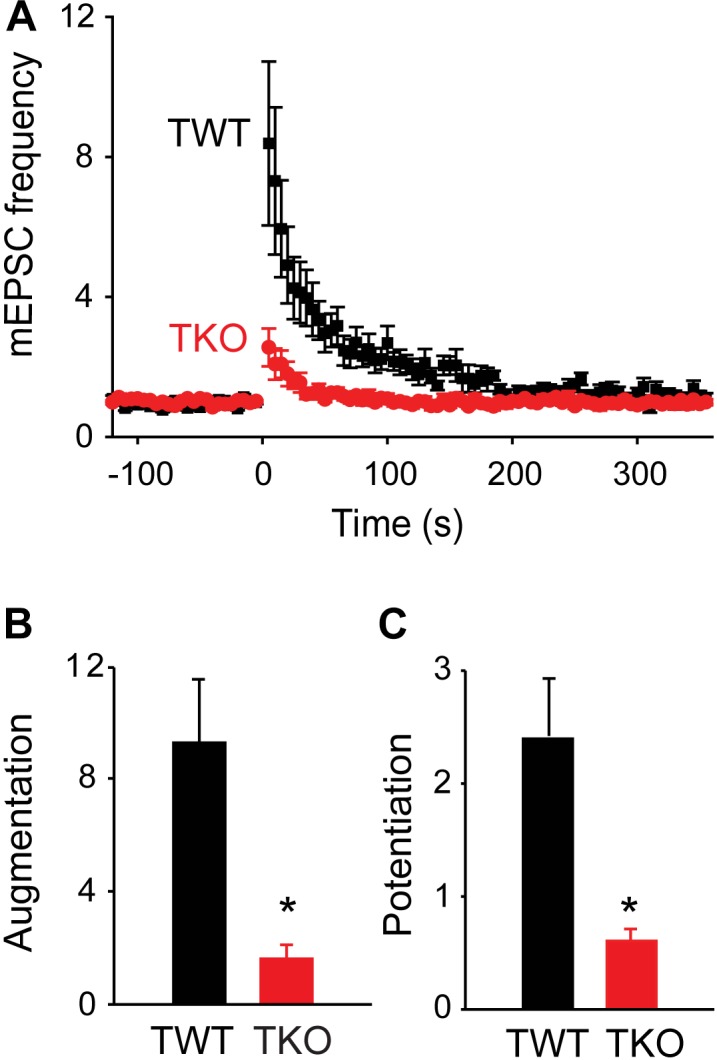
Role of synapsins in augmentation and potentiation. **(A)** Time course of changes in normalized mEPSC frequency produced by electrical activity (50 Hz, 2 s stimulation) in TWT (black) and TKO (red) neurons. Points indicate mean values of mEPSC frequency (*n* = 7), normalized to baseline values measured prior to the stimulus train, and error bars indicate ± SEM. **(B)** Peak amount of augmentation of mEPSC frequency, calculated from exponential fits to the data in panel **(A)**, in TWT (black) and TKO (red) neurons. **(C)** Amount of potentiation of mEPSC frequency in TWT (black) and TKO (red) neurons. Values in panles **(B,C)** indicate means and error bars indicate ± SEM, while asterisks indicate significant differences (*p* < 0.05) between TWO and TKO.

To evaluate the role of synapsins, we compared augmentation and potentiation in neurons from synapsin TKO and TWT mice. In TKO neurons, both augmentation and potentiation were significantly reduced (Figure 2A, *n* = 7). The amplitude of the augmentation component was reduced by 82.6% (to a 162% ± 50% increase in mEPSC frequency; *p* = 0.006, Student’s *t*-test; Figure 2B), while the amplitude of the potentiation component was reduced by 74.6% (to a 62.2% ± 12% increase in mEPSC frequency; *p* = 0.01, Student’s *t*-test; Figure 2C) The kinetics of the remaining augmentation (time constant = 18.0 ± 4.6 s, *n* = 7) and potentiation (time constant = 149 ± 55 s, *n* = 7) were similar to control values (*p* = 0.83, Student’s *t*-test). The substantial attenuation of augmentation and potentiation observed in TKO neurons indicates that synapsins play important roles in both of these types of short-term synaptic plasticity.

**FIGURE 3:**
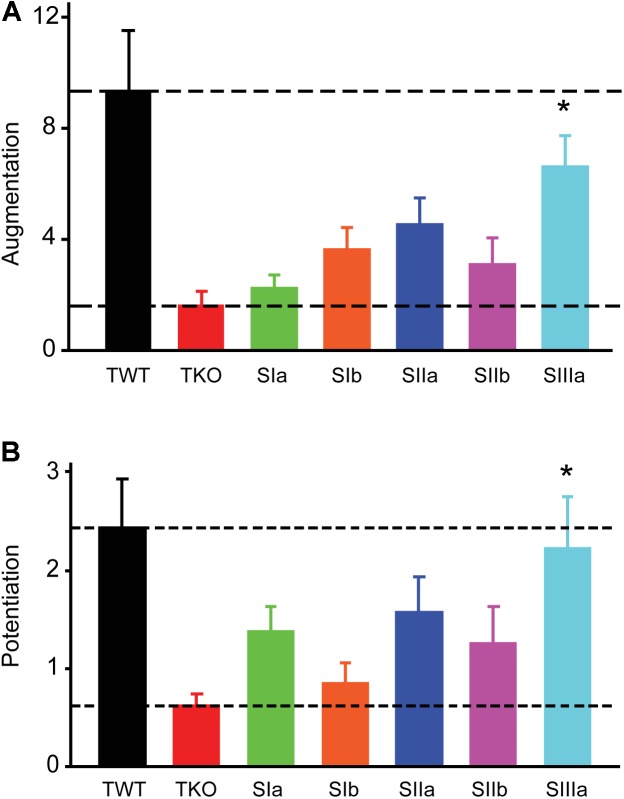
Rescue of augmentation and potentiation defects in TKO neurons by synapsin isoforms. **(A)** Amplitude of augmentation in TWT (black) and TKO (red) neurons, as well as in TKO neurons expressing indicated synapsin isoforms. **(B)** Amplitude of potentiation in the same conditions shown in panel **(A)**. Values in panels **(A,B)** indicate means, determined as in Figure 2, error bars indicate ± SEM, and asterisks indicate significant differences compared to values measured in TKO neurons (lower dashed lines).

In TKO mice, all synapsin isoforms are eliminated. To identify the specific synapsin isoforms involved in augmentation and potentiation, we determined which isoforms could rescue the reductions in these forms of short-term plasticity observed in TKO neurons. For this purpose, we infected TKO neurons with lentivirus encoding GFP-tagged versions of five synapsin (syn) isoforms: synIa, Ib, IIa, IIb and IIIa (Gitler et al., 2008). GFP-tagged synapsins have been shown to function normally in terms of synaptic targeting (Gitler et al., 2004a,b), phosphorylation by protein kinases (Chi et al., 2003), and their ability to rescue both glutamatergic (Gitler et al., 2008) and GABAergic (Song and Augustine, 2016) synaptic transmission in synapsin TKO neurons. Synapsin isoforms differed in their ability to rescue synaptic augmentation (Figure 3A) and potentiation (Figure 3B). In TKO neurons expressing synIIIa, the amplitude of augmentation was increased (*p* < 0.001, *t* = 3.86; *n* = 8), as was the amplitude of potentiation (*p* = 0.001, *t* = 3.45; *n* = 8). However, synIIIa did not fully restore augmentation or potentiation in TKO neurons to the levels measured in TWT neurons (TWT vs. TKO-synIIIa: *p* = 0.24 for augmentation and *p* = 0.14 for potentiation). In contrast, the amplitude of augmentation was not significantly rescued in TKO neurons expressing synIa (*p* = 0.62, *t* = 0.50; *n* = 10), synIb (*p* = 0.13, *t* = 1.50; *n* = 7), synIIa (*p* = 0.03, *t* = 2.18; *n* = 7), or synIIb (*p* = 0.22, *t* = 1.21; *n* = 11). Similarly, the amplitude of potentiation was not significantly rescued by synIa (*p* = 0.14, *t* = 1.52; *n* = 10), synIb (*p* = 0.68, *t* = 0.41; *n* = 7), synIIa (*p* = 0.08, *t* = 1.75; *n* = 7), or synIIb (*p* = 0.20, *t* = 1.29; *n* = 11). These results indicate that synapsins play an important role in augmentation and potentiation, with the synapsin IIIa isoform having the greatest ability to rescue both the augmentation and potentiation phenotypes of TKO neurons.

### Differential Roles of PKA and PKC

**FIGURE 4:**
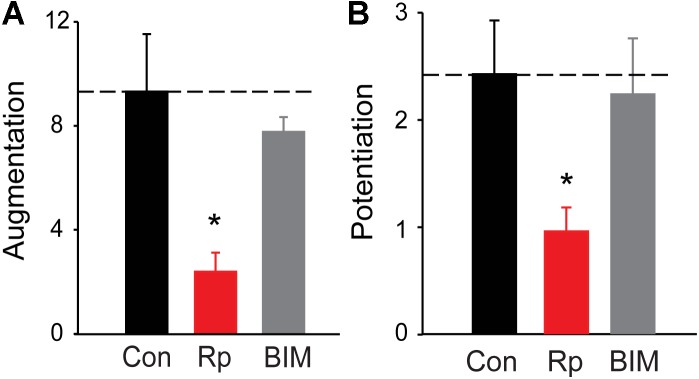
Roles of PKA and PKC in augmentation and potentiation. **(A)** Amplitude of augmentation measured in TWT neurons in control conditions (black), as well as in the presence of the PKA inhibitor Rp-cAMP (red) and the PKC inhibitor BIM (gray). **(B)** Amplitude of potentiation the same conditions indicated in panel **(A)**. Values indicate means, determined as in Figure 2, error bars indicate ± SEM, and asterisks indicate significant differences compared to values measured in control conditions (dashed lines).

To define the potential role of protein kinase(s) in augmentation and potentiation, we examined the effects of protein kinase inhibitors on augmentation and potentiation in TWT neurons. Because PKC has been implicated in augmentation and/or potentiation at a number of synapses (Beierlein et al., 2007; Korogod et al., 2007; Fioravante et al., 2011; Genc et al., 2014), we first tested bisindolylmaleimide (BIM; 0.5 μM). This drug inhibits PKC by blocking its ATP-binding site (Gassel et al., 2004) and has been shown to block PTP of evoked EPSCs at hippocampal CA3-CA1 synapses (Brager et al., 2003). However, we did not observe a significant reduction in either augmentation (Figure 4A; *p* = 0.34, *t* = −0.97; *n* = 4) or potentiation (Figure 4B; *p* = 0.68, *t* = −0.41; *n* = 4) of spontaneous glutamate release following treatment with BIM. This suggests that PKC is not involved in either augmentation or potentiation of spontaneous glutamate release in cultured hippocampal neurons. This conclusion is consistent with findings at hippocampal mossy fiber synapses (Wang et al., 2016) and also is consistent with the fact that PKC does not phosphorylate synapsins (Hilfiker et al., 2005).

All synapsin isoforms, including synIIIa, are known to be phosphorylated by PKA (Hosaka et al., 1999). Given the role of PKA in augmentation and/or potentiation at numerous synapses (Kuromi and Kidokoro, 2000; Alle et al., 2001; Fiumara et al., 2004), we next determined the involvement of PKA in synapsin-dependent augmentation and potentiation. We first examined the effects of Rp-cAMPS, a membrane-permeant cyclic AMP analog that binds to the regulatory subunit of PKA and prevents activation of the catalytic subunit that phosphorylates PKA substrates (de Wit et al., 1982; Van Haastert et al., 1984; Rothermel and Parker Botelho, 1988; Dostmann et al., 1990). Bath application of Rp-cAMPS (25 μM) reduced the amplitude of both augmentation (by 74.4%; *p* = 0.0012, *t* = −3.65; *n* = 8) and potentiation (by 60.4%; *p* = 0.02, *t* = −2.47; *n* = 8), as shown in Figure 4. The degree of reduction of both augmentation and potentiation by Rp-cAMPS is roughly similar to the degree of reduction produced by loss of synapsins (Figure 2). These results suggest that activation of PKA is important for synapsin-dependent synaptic plasticity.

**FIGURE 5:**
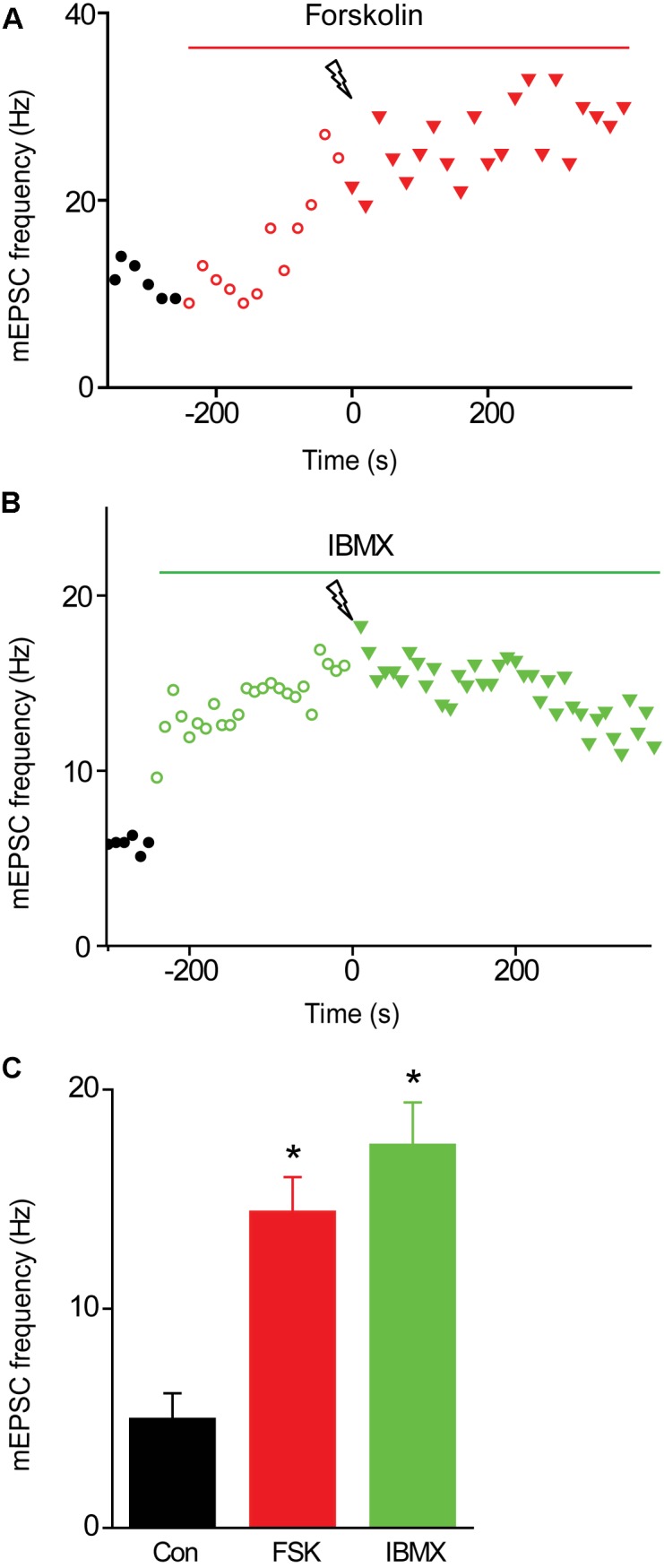
Effects of elevating presynaptic cAMP levels on mEPSC frequency in TWT neurons. **(A)** Time course of changes in normalized mEPSC frequency (red symbols) produced by application of forskolin (10 μM) during time indicated by red bar. Electrical stimulation (50 Hz, 2 s) was applied at time = 0. **(B)** Time course of changes in mEPSC frequency (green symbols) produced by application of IBMX (0.5 mM) during time indicated by green bar. Electrical stimulation (50 Hz, 2 s) was applied at time = 0. **(C)** Mean values of mEPSC frequency measured in control conditions (black), as well as in the presence of forskolin (FSK; red) and IBMX (green). Values indicate means, error bars indicate ±SEM, and asterisks indicate significant differences compared to values measured in control conditions.

**FIGURE 6:**
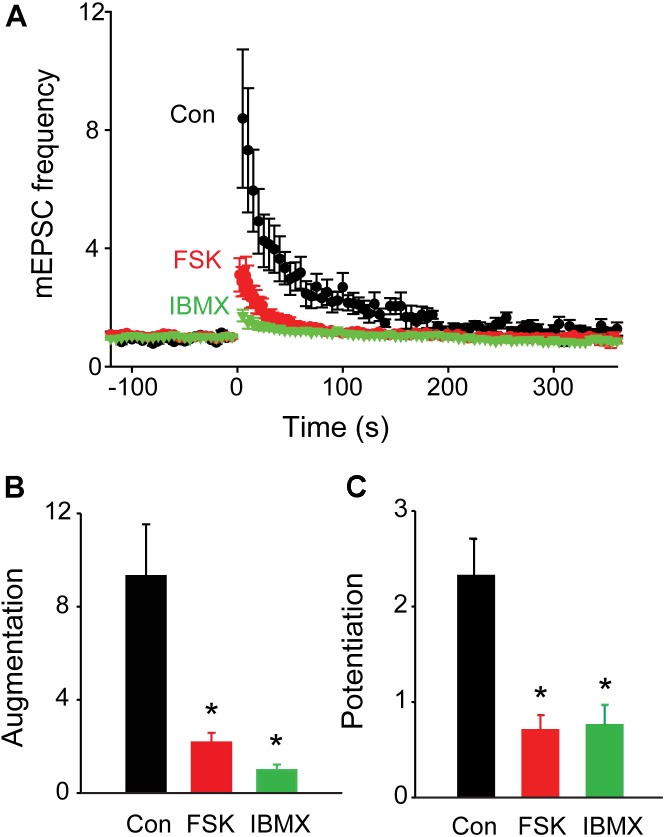
Occlusion of synaptic plasticity following elevation of presynaptic cAMP levels. **(A)** Time course of changes in normalized mEPSC frequency produced by electrical activity (50 Hz, 2 s stimulation) in TWT neurons in control conditions (black), as well as in the presence of forskolin (FSK; 10 μM; red) and IBMX (green). Points indicate mean values of mEPSC frequency (*n* = 5), normalized to baseline values measured prior to the stimulus train, and error bars indicate ±SEM. **(B)** Peak amount of augmentation of mEPSC frequency, calculated from exponential fits to the data in panel **(A)**, in control (black), forskolin (red), and IBMX (green). **(C)** Amount of potentiation of mEPSC frequency in same conditions described in panel **(B)**. Values in panels **(B,C)** indicate means and error bars indicate ±SEM, while asterisks indicate significant differences between control and forskolin or control and IBMX.

We next examined the effects of activating PKA by elevating intracellular cAMP concentration. We began by treating the cultured neurons with forskolin, which elevates cAMP concentration by activating adenylyl cyclase (Seamon et al., 1983). Application of forskolin (10 μM) caused a time-dependent increase in mEPSC frequency (Figure 5A) which at its peak increased mEPSC frequency by approximately 3-fold over the basal level (Figure 5C). Thus, increasing PKA activity potentiates spontaneous transmitter release, as reported previously at many synapses (Yoshihara et al., 2000; Sakaba and Neher, 2001; Kaneko and Takahashi, 2004; Miura et al., 2012). Delivery of a train of stimuli (50 Hz, 2 s) in the presence of forskolin reduced augmentation and potentiation (Figure 5A, solid triangles and Figure 6A, red points). On average, the amplitude of augmentation was reduced by 73.5% (Figure 6B; *p* = 0.004, *t* = −4.62; *n* = 7) and potentiation was reduced by 69.2% (Figure 6C; *p* = 0.02, *t* = −2.51; *n* = 7). Thus, elevation of cAMP levels enhanced spontaneous transmitter release and occluded both the augmentation and potentiation of spontaneous release produced by electrical activity.

Elevation of cAMP levels by treatment with the inhibitor IBMX, which blocks the phosphodiesterase responsible for degradation of cAMP (Deth and Lynch, 1981; Leroy et al., 2008), produced similar effects. Application of IBMX (0.5 mM) caused a time-dependent increase in mEPSC frequency (Figure 5B); at its peak, IBMX increased mEPSC frequency by approximately 4-fold over basal levels (Figure 5C) and reduced the increase in mEPSC frequency evoked by a train of electrical stimuli (Figure 5B, solid triangles and Figure 6A, green points). The mean reductions in the amplitude of augmentation (87.8%; *p* = 0.03, *t* = −3.73; *n* = 7) and potentiation (67.5%; *p* = 0.009, *t* = −2.78; *n* = 7) were comparable to the effects of forskolin treatment (Figures 6B,C). Taken together, these results suggest that activation of PKA by cAMP is involved in both forms of short-term synaptic plasticity.

### Synapsins as PKA Substrates During Synaptic Plasticity

**FIGURE 7:**
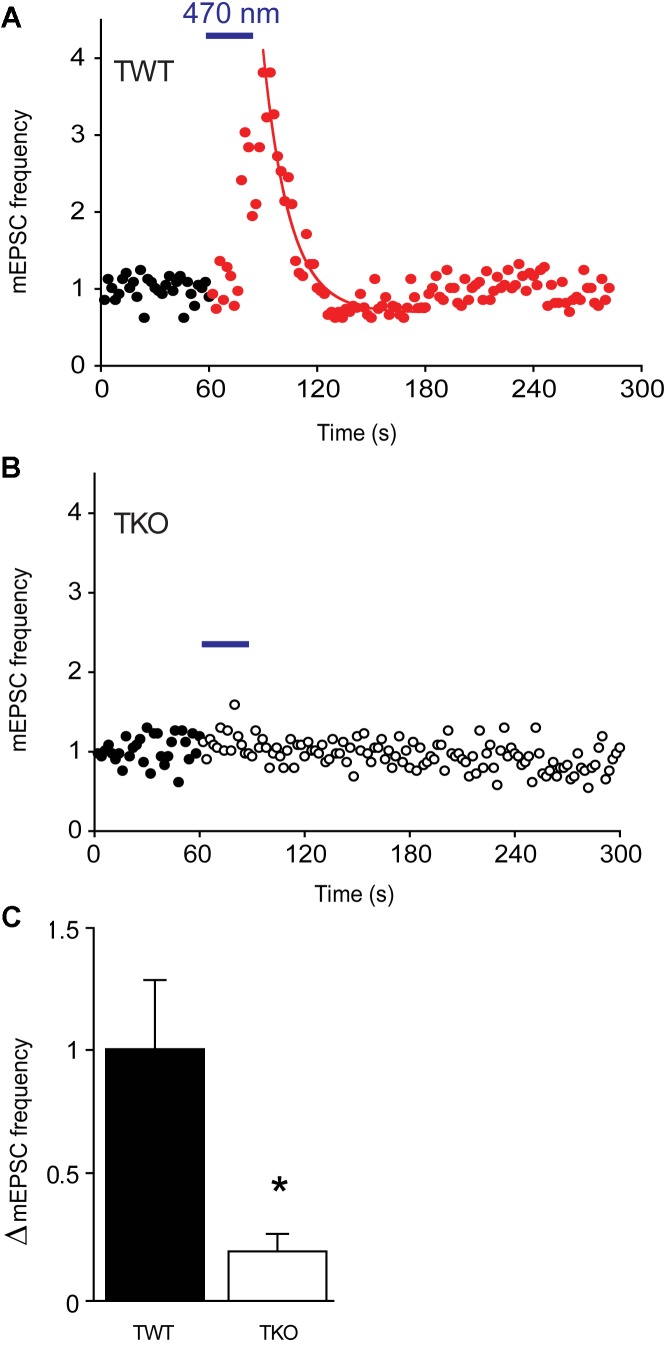
Changes in mEPSC frequency produced by optogenetic elevation of presynaptic cAMP levels. **(A)** Time course of normalized mEPSC frequency in response to illumination (470 nm light, 30 s at blue bar) of a TWT neuron expressing opto-β2-AR. Red points indicate mEPSC frequency, normalized to baseline values, measured following the start of the light flash. Smooth curve indicates an exponential fit to the decay of mEPSC frequency following the end of the light flash. **(B)** Same for a TKO neuron expressing opto-β2-AR. Open symbols indicate normalized mEPSC frequency measured following the start of the light flash. **(C)** Mean increase in mEPSC frequency produced by activation of opto-β2-AR in WT (black) and TKO (white) neurons. Error bars indicate ±SEM, while asterisks indicate significant differences.

To define the temporal relationship between PKA activation and synaptic plasticity, we next made a time-resolved jump in cAMP concentration within the presynaptic terminal. For this purpose, we expressed in the cultured neurons opto-β2-AR, a light-sensitive, chimeric G-protein coupled receptor that produces a rapid elevation in cAMP concentration in response to blue light (Airan et al., 2009). In TWT neurons expressing opto-β2-AR, a brief light flash (470 ± 20 nm, 30 s duration) produced a transient increase in mEPSC frequency (Figure 7A). mEPSC frequency gradually increased during the light flash, reached a peak almost immediately after the end of the flash, and exponentially decayed back to baseline levels afterward. The mean increase in mEPSC frequency was 97.3 ± 28.8% (Figure 7C; *n* = 6) and the time constant for decay of mEPSC frequency after the flash was 35.8 ± 10.6 s (*n* = 6), a decay time course that is intermediate between those of augmentation and potentiation. In TKO neurons expressing opto-β2-AR, the same light flash produced a significantly smaller (*p* = 0.03, Student’s *t*-test, *n* = 3) increase in mEPSC frequency (Figure 7B), with mean increase of 19.7 ± 6.8% (Figure 7C; *n* = 3). Thus, a rapid jump in presynaptic cAMP concentration was capable of generating an increase in spontaneous glutamate release that resembles augmentation and potentiation. Further, similar to augmentation and potentiation, this increase depends upon synapsins.

**FIGURE 8:**
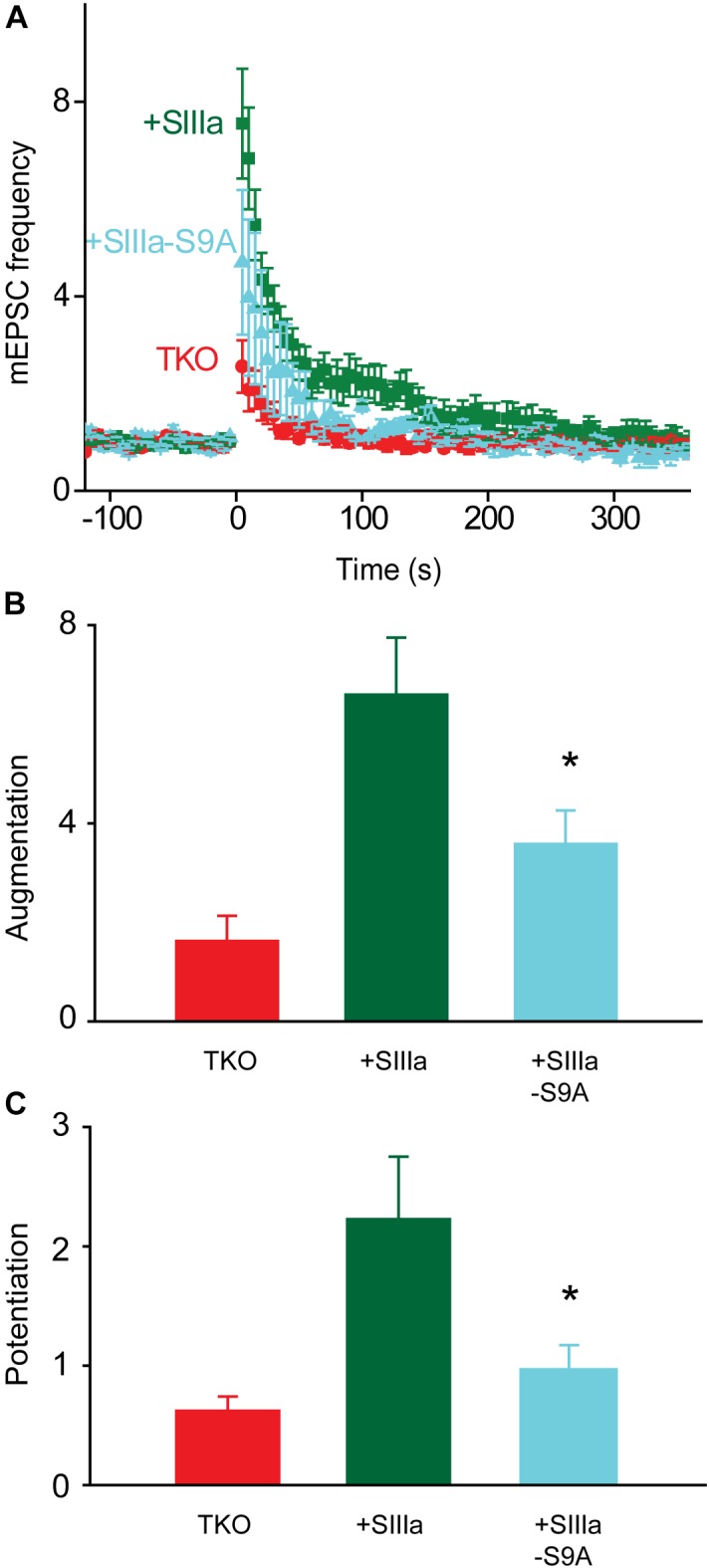
PKA regulation of synapsin IIIa rescue of TKO phenotype. **(A)** Time course of changes in mEPSC frequency produced by electrical activity (50 Hz, 2 s stimulation) in TKO neurons (red) and in TKO neurons expressing wild-type synapsin IIIa (+SIIIa; green) or phosphorylation-deficient synapsin IIIa S9A (+SIIIa-S9A; cyan). Points indicate mean values of mEPSC frequency (*n* = 5), normalized to baseline values measured prior to the stimulus train, and error bars indicate ±SEM. **(B)** Peak amount of augmentation of mEPSC frequency, calculated from exponential fits to the data in panel **(A)**, in TKO neurons (red) and in TKO neurons expressing wild-type synapsin IIIa (green) or phosphorylation-deficient synapsin IIIa S9A (cyan). Values in panels **(B,C)** indicate means and error bars indicate ± SEM, while asterisks indicate significant differences compared to TKO + SynIIIa neurons.

Finally, we asked which sites on synapsins are phosphorylated by PKA to produce augmentation and potentiation. Synapsin IIIa, the isoform most effective in rescuing augmentation and potentiation in TKO neurons (Figure 3), contains a known PKA phosphorylation site (serine 9). We mutated this serine into non-phosphorylatable alanine to prevent PKA from phosphorylating this residue. Expression of synapsin IIIa in TKO neurons rescued both augmentation and potentiation (Figure 8A), as indicated in Figure 3. However, the rescue of augmentation and potentiation in TKO neurons by synapsin IIIa was greatly reduced by the synIIIa-S9A mutant (Figure 8A). This was true for both the augmentation (Figure 8B; 60.6% reduction; *p* = 0.03, *t* = −2.37; *n* = 6) and potentiation (Figure 8C; 77.5% reduction; *p* = 0.01, *t* = −2.73; *n* = 4). This indicates that the PKA phosphorylation site of synapsin IIIa is critical for rescue of augmentation and potentiation in TKO neurons. The same mutation in the PKA phosphorylation site of synapsin IIa also eliminated the partial rescue produced by synIIa (data not shown). Taken together, we conclude that synapsin IIIa is important for augmentation and potentiation, specifically by serving as a substrate for PKA to transiently enhance the rate of spontaneous glutamate release in response to repetitive presynaptic activity.

## Discussion

Augmentation and potentiation are two forms of short-term plasticity that enhance neurotransmitter release for seconds to minutes following a bout of presynaptic activity. Here we have examined the molecular signaling underlying these two forms of plasticity in cultured hippocampal neurons. We found that PKA, but not PKC, is involved in regulation of both augmentation and potentiation of spontaneous glutamate release. Further, this kinase seems to act by phosphorylating synapsins, specifically the synapsin IIIa isoform.

### Roles of Protein Kinases in Augmentation and Potentiation

While it is well-established that both augmentation and potentiation are triggered by transient rises in presynaptic calcium concentration, the involvement of downstream protein kinase signaling in these forms of plasticity is much less clear. While PKC clearly plays a role in potentiation at the glutamatergic calyx of Held synapse (Korogod et al., 2007; Fioravante et al., 2011), the role of this kinase in potentiation of hippocampal synapses is uncertain. Both our data (Figure 4B) and those of Wang et al. (2016) indicate that potentiation of glutamate release onto hippocampal pyramidal cells does not require PKC. However, PKC may be involved in potentiation at glutamatergic synapses onto hippocampal interneurons (Alle et al., 2001). Thus, there seem to be clear differences in the importance of PKC for potentiation at different synapses, even within the same brain area.

We have established several lines of evidence implicating PKA in potentiation of excitatory synapses onto hippocampal pyramidal cells. First, a PKA inhibitor reduced potentiation (Figure 4B). Second, two different pharmacological treatments that elevate cAMP levels enhanced spontaneous glutamate release (Figure 5) and occluded potentiation (Figures 6A,C). Third, transient optogenetic elevation of cAMP levels evoked a transient, potentiation-like enhancement of spontaneous release (Figures 7A,C). Finally, deletion of a PKA phosphorylation site in synapsin IIIa reduced the ability of this isoform to rescue the loss of potentiation observed in synapsin TKO neurons (Figure 8). Thus, we conclude that PKA is important for potentiation of spontaneous release at these synapses. This is consistent with observations that inhibitors of PKA reduce potentiation of glutamatergic synapses onto hippocampal interneurons (Alle et al., 2001) and at excitatory synapses of *Helix* (Fiumara et al., 2007) and *Aplysia* (Khoutorsky and Spira, 2009). Although PKA is not directly activated by calcium, adenylyl cyclase is activated by Ca^2+^/calmodulin (Hanoune and Defer, 2001; Wang and Zhang, 2012) and this could allow PKA to be activated during a tetanus.

Remarkably, we found that PKA also is important for augmentation of spontaneous glutamate release at excitatory synapses onto hippocampal pyramidal cells. While there have been few studies of the molecular mechanisms of augmentation, to date most analyses suggest that augmentation results from calcium directly binding to calcium-regulated proteins such as munc13 (Rosenmund et al., 2002; Gioia et al., 2016) rather than from kinase-mediated signaling. Thus, our findings open a new window into the signaling processes underlying augmentation. For example, our observation that elevation of presynaptic cAMP levels alone causes an enhancement of transmitter release that lasts longer than augmentation (Figure 7A) suggests that the decay of augmentation could be accelerated by an activity-dependent decay in the levels of synapsin phosphorylation. A plausible hypothesis for such a mechanism would be calcium-dependent activation of the protein phosphatase, calcineurin, which is able to dephosphorylate synapsins (King et al., 1984).

### Synapsin Isoforms and Synaptic Plasticity

Synapsins are known to control synaptic vesicle mobilization during periods of intense synaptic activity, such as the type of activity that elicits augmentation and potentiation. Further, phosphorylation regulates the binding affinity of synapsins for synaptic vesicles and cytoskeletal elements (Greengard et al., 1993; Hilfiker et al., 1999; Hosaka et al., 1999; Cesca et al., 2010). Given the roles of protein phosphorylation in both augmentation and potentiation, it is therefore possible that synapsins could serve as downstream targets of protein kinase signaling during short-term synaptic plasticity. Consistent with this possibility, previous work has shown that potentiation is reduced both by genetic deletion of synapsins at mouse excitatory synapses (Rosahl et al., 1995; Valente et al., 2012) and by antibody neutralization of synapsin at *Aplysia* inhibitory synapses (Humeau et al., 2001). We have extended these findings by showing that both augmentation and potentiation are almost completely eliminated at excitatory hippocampal synapses of synapsin TKO neurons (Figure 2). Further, we have systematically evaluated the ability of each synapsin isoform to support these forms of short-term synaptic plasticity (Figure 3) and the role of PKA phosphorylation in rescue of augmentation and potentiation by synapsin IIIa (Figures 7, 8).

We found that not all synapsin isoforms are involved in augmentation and potentiation of excitatory transmission: only synapsin IIIa was capable of significantly rescuing these forms of synaptic plasticity in synapsin TKO neurons. This extends previous work indicating that synapsin isoforms differ in their physiological functions (Song and Augustine, 2015). Our finding that synapsin IIa partially rescued augmentation and potentiation, an effect that did not reach statistical significance, is consistent with a previous study showing a partial loss of potentiation in synapsin II knock-out mice (Rosahl et al., 1995). Our results extend the earlier finding by indicating that the loss of synapsin IIa, rather than synapsin IIb, is likely responsible for the defect in potentiation. We found that synapsin IIIa had the greatest ability to rescue short-term plasticity, almost completely rescuing potentiation and largely rescuing augmentation (Figure 3). While synapsin IIIa is predominantly expressed during early neuronal development (Ferreira et al., 2000), it is also known to regulate neurotransmitter release in more mature neurons (Feng et al., 2002; Kile et al., 2010; Song and Augustine, 2016).

While mutation of the PKA phosphorylation site, serine 9, reduced the ability of synapsin IIIa to rescue augmentation and potentiation in TKO neurons, this mutation did not completely lower these forms of synaptic plasticity down to the levels observed in control TKO neurons. This could indicate a role for other phosphorylation sites in the regulation of synapsin IIIa function during augmentation and potentiation. In addition to this PKA phosphorylation site, synapsin IIIa also possesses a unique MAPK phosphorylation site within its J domain. Given the role of MAPK phosphorylation in regulating vesicle trafficking (Chi et al., 2003) and potentiation (Schenk et al., 2005; Khoutorsky and Spira, 2009; Giachello et al., 2010) during synaptic activity, it is possible that this kinase could also phosphorylate synapsin IIIa during augmentation and potentiation. Other evidence implicates still other protein kinases, such as calcium/calmodulin-dependent protein kinases (Jin and Hawkins, 2003; Fiumara et al., 2007, but see Malinow et al., 1988; Stevens et al., 1994). Thus, it is possible that multiple protein kinases regulate augmentation and potentiation by phosphorylating synapsins, with the complement of relevant kinases likely to vary according to the type of synapse as well as the amount of synaptic activity (Chi et al., 2003; Yamagata and Nairn, 2015).

Phosphorylation of synapsin I by PKA is a key regulator of synaptic vesicle exocytosis and recycling (Chi et al., 2003; Menegon et al., 2006). However, we found that synapsin Ia only modestly rescued potentiation, an effect that did not reach statistical significance, and did not rescue augmentation at all. Further, synapsin Ib could not rescue either form of synaptic plasticity. These results are consistent with a report that potentiation is normal in the hippocampal CA1 region of synapsin I KO mice (Rosahl et al., 1995). Our results also fit with the observation of Valente et al. (2012) that loss of synapsin I causes a partial loss of potentiation in cultured hippocampal neurons, an effect that was rescued by synapsin Ia. Our results are also consistent with the observation that overexpression of a *Helix* synapsin resembling synapsin I has no effect on augmentation (Fiumara et al., 2007).

### Mechanisms of Synapsin Action in Synaptic Plasticity

As described in the “Introduction” section, multiple mechanisms are involved in the enhancement of neurotransmitter release during augmentation and potentiation. It is not yet clear which, if any, of these mechanisms involve PKA and synapsin IIIa. PKA-mediated phosphorylation could allow synapsin IIIa to dissociate from synaptic vesicles within the reserve pool, thereby mobilizing these vesicles to enhance their availability to participate in glutamate release (Kuromi and Kidokoro, 2000). This model is attractive because PKA-mediated phosphorylation of synapsin IIIa controls both augmentation and potentiation, both of which ultimately depend upon mobilization of synaptic vesicles from the reserve pool. However, synapsin IIa is the only isoform that can maintain vesicles within the reserve pool; synapsin IIIa has no significant ability rescue the defect in vesicle reserve pool size observed in TKO neurons (Gitler et al., 2008). The fact that synapsin IIIa can rescue augmentation and potentiation in TKO neurons, without rescuing the reserve pool, argues that the regulatory role of synapsins in short-term synaptic plasticity does not arise from an effect on the reserve pool. Alternatively, it is known that synapsins can produce activity-stimulated increases in the number of active release sites (Humeau et al., 2007) as well as having other effects on the readily releasable pool of synaptic vesicles (Hilfiker et al., 1998; Humeau et al., 2001; Medrihan et al., 2013; Song and Augustine, 2016). Further work will be required to determine whether these or other mechanisms permit PKA-mediated phosphorylation of synapsins to regulate neurotransmitter release during augmentation and potentiation.

## Ethics Statement

The procedures used to maintain and use mice were approved by Duke University Institutional Animal Care and Use Committee, Biopolis Institutional Animal Care and Use Committee, and Nanyang Technological University Institutional Animal Care and Use Committee.

## Author Contributions

QC and GA designed the experiments. QC and SHS performed the experiments and analyzed the data. All authors wrote the paper.

## Conflict of Interest Statement

The authors declare that the research was conducted in the absence of any commercial or financial relationships that could be construed as a potential conflict of interest.
